# Assessment of the Efficacy of Subgingivally Delivered Liquorice Gel on Clinical Parameters and Prostaglandin E2 Levels in Chronic Periodontitis: A Clinico-Biochemical Study

**DOI:** 10.7759/cureus.76781

**Published:** 2025-01-02

**Authors:** Bipin Kumar Yadav, Diksha Gupta, Rajesh Kumar Thakur, Jatin Chauhan, Rahul Mishra, Ajai Kumar

**Affiliations:** 1 Department of Periodontology, Faculty of Dental Sciences, Uttar Pradesh University of Medical Sciences, Etawah, IND; 2 Department of Biochemistry, Uttar Pradesh University of Medical Sciences, Etawah, IND

**Keywords:** gingival crevicular fluid, liquorice gel, local drug delivery agent, prostaglandin e2 levels, scaling and root planing

## Abstract

Background: Liquorice, also known as "mulaithi" in North India, has shown anti-inflammatory, antimicrobial, and anti-adherence effects that can prevent and treat various periodontal diseases.

Aim: This study aims to assess the efficacy of subgingivally delivered liquorice gel on clinical parameters and prostaglandin E2 (PGE2) levels in chronic periodontitis.

Materials and methods: A split-mouth, single-blind, prospective, parallel, randomized controlled clinical trial was conducted. Seventeen patients diagnosed with moderate chronic periodontitis with bilateral nearly symmetrical sites having a pocket depth of ≥4 mm but ≤6 mm were included in the study. A total of 60 sites were randomly divided into two groups, with 30 sites in each group: Group A, scaling and root planing (SRP) with subgingival delivery of liquorice gel, and Group B, SRP with subgingival delivery of placebo gel. The clinical parameters, plaque index (PI), gingival index (GI), probing pocket depth (PPD), clinical attachment level (CAL), and gingival crevicular fluid (GCF), samples were collected at baseline, 15th day, and 30th day after gel placement. The collected GCF samples were subjected to biochemical analysis of PGE2 levels using an ELISA kit. The Mann-Whitney U and Wilcoxon signed-rank tests were performed for intergroup and intragroup comparisons.

Results: Statistically significant improvements in clinical and biochemical parameters were observed in both groups over time. Intergroup comparison of PI and GI showed statistically non-significant differences at all time intervals. Similarly, intergroup comparisons of PPD, CAL, and PGE2 levels showed statistically non-significant differences at baseline and 15th day. However, on the 30th day, PPD, CAL, and PGE2 levels revealed statistically significant differences between both groups. Our result corroborated that Group A, tested with liquorice gel, showed better results than Group B.

Conclusion: Liquorice gel can be used as an effective local drug delivery agent as an adjunct to SRP for treating chronic periodontitis.

## Introduction

Periodontitis is a complex, inflammatory disease of periodontium resulting in the deterioration of periodontal tissue and alveolar bone. The disease is associated with microbial plaque and contributing factors, including exaggerated immune response, genetic predisposition, and environmental factors [1].

The management of periodontitis concentrates on eradicating supragingival and subgingival plaque and calculus, intending to restore the healthy state of oral tissues. However, the complete elimination of periodontal pathogens may not be achieved by nonsurgical procedures alone due to the unique characteristics of pathogens that reside in deep and inaccessible areas. Therefore, it appears reasonable to combine mechanical periodontal therapy with the administration of systemic or local chemotherapeutic agents.

To avoid undesirable adverse effects of systemic antimicrobials, Goodson et al. first proposed the concept of controlled local drug delivery of drugs in periodontal pockets for treating periodontitis [2].

Numerous pharmacological agents have already been used, including doxycycline, minocycline, tetracycline, metronidazole, chlorhexidine, alendronate, simvastatin, and others. Recently, herbal extracts containing phytochemicals responsible for the desired anti‑inflammatory and antimicrobial effects have been used in modern medicine [3].

Liquorice (*Glycyrrhiza glabra*), commonly known as sweet wood, is indigenous to the Mediterranean and a few areas of Asia. The term *Glycyrrhiza* originates from the ancient Greek words glycos, meaning sweet, and rhiza, meaning root, also known as "mulaithi" in north India. The bioactive components found in liquorice have shown anti-inflammatory [4], antimicrobial [5], and anti-adherence [6] effects that can help prevent and treat periodontal disease as well. Key bioactive ingredients in liquorice extract are glycyrrhizin, glabridin, licoricidin, licochalcone A, and licorisoflavan A.

In vitro, studies suggested that liquorice prevents cyclooxygenase activity, potentially leading to inhibition of various components in the inflammatory cascade, including prostaglandin E2 (PGE2) and reactive oxygen species at the inflammatory site and, thus, may reduce the inflammatory markers at the site of infection [7].

PGE2, an arachidonic acid metabolite, induces vasodilatation, increases vascular permeability, and has multiple effects on different cell types in the periodontium [8]. A reduction in PGE2 level in gingival crevicular fluid (GCF) indicates a reduction in inflammation and, thus, reduces bone resorption. Therefore, it can serve as a valuable indicator of periodontitis progression.

Based on this background, the present study will evaluate the efficacy of subgingivally delivered liquorice gel as an adjunct to scaling and root planing (SRP) on clinical parameters and prostaglandin E2 level in chronic periodontitis.

## Materials and methods

A prospective, randomized, comparative, single-blind, split-mouth study was conducted in the Department of Periodontology, Faculty of Dental Sciences, Uttar Pradesh University of Medical Sciences, Saifai, Etawah, UP, India. The study was conducted over 15 months, from January 2023 to April 2024, after getting approval from the Uttar Pradesh University of Medical Sciences Ethical Committee (approval number: 38/2022-23) on 19 patients with 64 sites having moderate chronic periodontitis. Out of these, two patients with four sites were lost to follow-up. The written informed consent was obtained from each patient after explaining the treatment procedure before the commencement of the study. The sample size was calculated based on variation in gingival score change from baseline to last follow-up in two study groups [9]. Type II errors may be termed as "statistical power," i.e., having a 10% type II error is the same as the study had 90% "power." Therefore, based on this formula, 30 sites in each group were sufficient for conducting the study with a power analysis of 90%. The study was registered at ctri.nic.in with CTRI no. CTRI/2024/05/067674.

Inclusion and exclusion criteria

The inclusion criteria include patients aged 25 to 50 years with moderate chronic periodontitis with bilateral nearly symmetrical sites with a pocket depth of ≥4 mm but ≤6 mm. Systemically healthy patients were also enrolled in the study. Written informed consent was obtained from each patient after explaining the treatment procedure before the commencement of the study.

Medically compromised patients, smokers, pregnant or lactating mothers, and patients who had undergone periodontal therapy or used antibiotics/analgesics/anti-inflammatory drugs in the last six months were excluded from the study.

Study design

**Figure 1:**
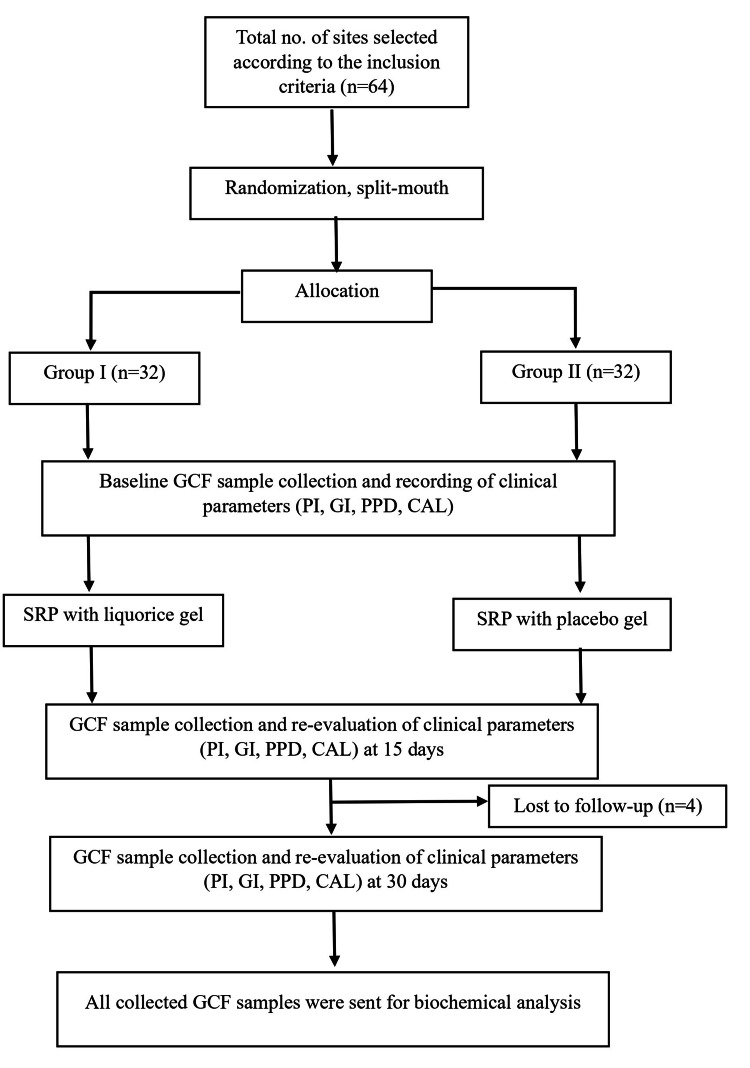
Flowchart of the study n: selected sites, GCF: gingival crevicular fluid, PI: plaque index, GI: gingival index, PPD: probing pocket depth, CAL: clinical attachment level, SRP: scaling and root planing

A split-mouth, parallel, randomized, controlled clinical trial with single blinding was conducted. Sites meeting the inclusion criteria were randomly divided into two groups using the flip-coin method. Group A underwent SRP with the subgingival placement of liquorice gel, prepared in the Department of Pharmacognosy with a 0.1% liquorice extract concentration. Group B received SRP with the subgingival placement of a placebo gel, also prepared in the Department of Pharmacognosy, containing a polymeric base. A summarized representation of the study design is provided in Figure 1.

Parameters

The clinical and biochemical parameters were recorded at baseline (before SRP and gel placement), 15th day, and 30th day after gel placement. Clinical parameters were plaque index (PI) by Silness and Loe [10], measured using a two-tone disclosing solution (AlphaPlac®); gingival index (GI) by Silness and Loe [11]; probing pocket depth (PPD), measured using a UNC-15 periodontal probe; and clinical attachment level (CAL), measured using a customized occlusal stent from the lower border of the stent to the base of the pocket. The biochemical parameter was PGE2 levels, measured using an ELISA kit (Elabscience® Co. Ltd., USCN, Wuhan).

Preparation of the liquorice extract

Liquorice root powder was mixed with 70% ethanol and left for 72 hours at ambient temperature. After 72 hours, filtration was done using Whitman’s filter paper. The resultant clear filtrate was dried in a hot air oven to obtain a uniform, semi-solid, dark brown-colored liquorice extract. A concentration of 0.1 mg/ml of ethanol extract of liquorice was utilized to develop a local drug delivery gel, with Carbopol 940 serving as a gel base as a mucoadhesive polymer.

Preparation of the liquorice gel

To prepare liquorice gel, 4 gm of Carbopol 940 was dissolved in 80 milliliters of water and constantly stirred for 30 minutes. Subsequently, 8 mg of liquorice extract was added and thoroughly mixed. The mixing was continued for one hour until a semisolid, homogeneous, light brown-colored gel was obtained. The pH of the formulated gel was 3.08, with adequate viscosity. The consistency of the formulations was investigated by pressing a small amount of gel between the index finger and thumb.

Preparation of the placebo gel

To prepare the placebo gel, 0.5 gm of Carbopol 940 was dissolved in 10 milliliters of distilled water and constantly stirred for 30 minutes. To ensure proper blinding of participants, the color additives were added to transform the initially prepared clear, transparent gel into a light brown-colored gel.

Procedure

**Figure 2:**
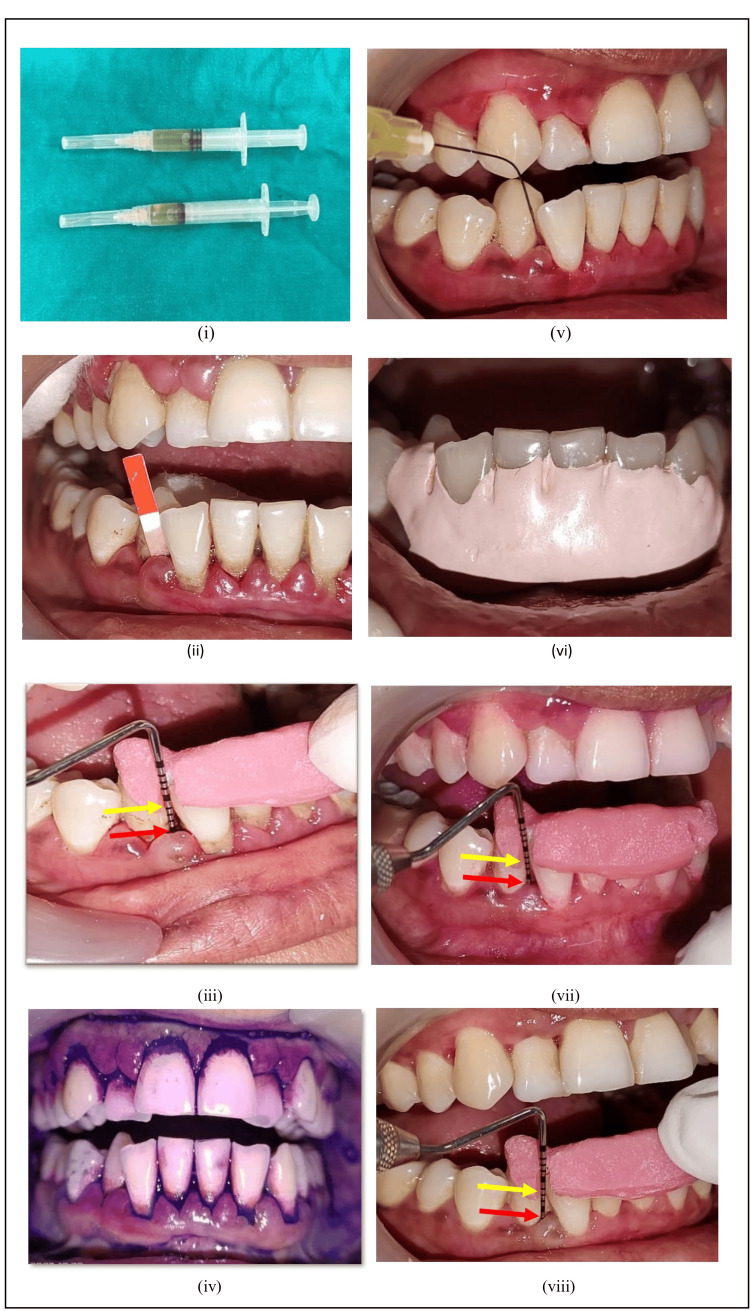
Clinical pictures of Group A (SRP with liquorice gel) (i) Prepared liquorice and placebo gel loaded in the syringe with 30 gauge single side-vent irrigation needle (Waldent); (ii) baseline GCF sample collected using periostrips from the selected site; (iii) PPD and CAL measured using a customized stent and periodontal probe; (iv) PI measured using disclosing solution; (v) After SRP, liquorice gel placed subgingivally using syringe and needle at the selected site; (vi) sites covered by periodontal dressing; (vii) reduction in PPD and CAL gain on 15th day after gel placement; (viii) reduction in PPD and CAL gain on 30th day. Yellow arrows indicate CAL and red arrows indicate PPD*. *GCF: gingival crevicular fluid, SRP: scaling and root planing, PPD: probing pocket depth, CAL: clinical attachment level, PI: plaque index

**Figure 3:**
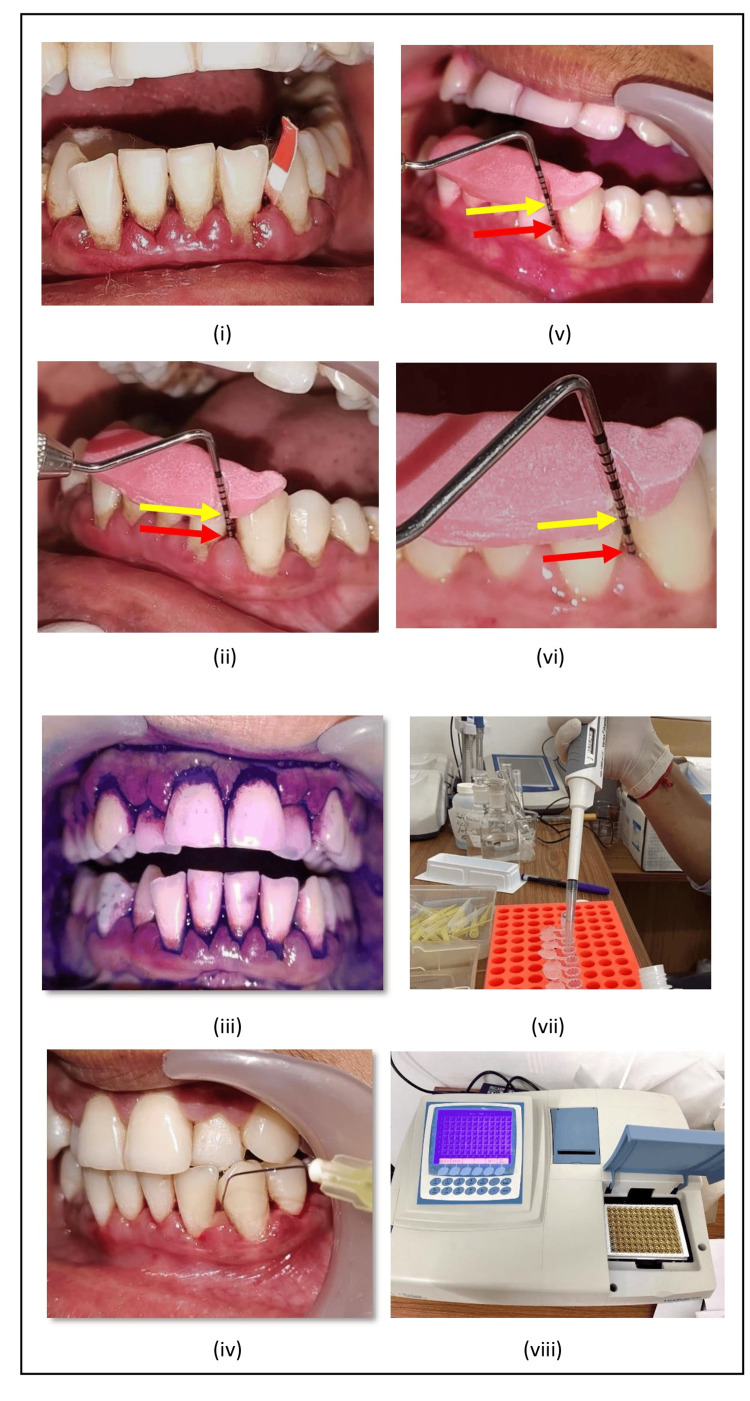
Clinical pictures of Group B (SRP with placebo gel) (i) Baseline GCF sample collected using periostrips from the selected site; (ii) PPD and CAL measured using a customized stent and periodontal probe; (iii) PI measured using disclosing solution; (iv) after SRP, placebo gel placed subgingivally using syringe and needle at the selected site; (v) reduction in PPD and CAL gain on 15th day after gel placement; (vi) reduction in PPD and CAL gain on 30th day; (vii) preparation of reagent for PGE2 level estimation using ELISA kit; (viii) recording of data using LisaScan® EM ELISA interpreter. Yellow arrows indicate CAL and red arrows indicate PPD. GCF: gingival crevicular fluid, SRP: scaling and root planing, PPD: probing pocket depth, CAL: clinical attachment level, PI: plaque index

After the selection of patients, a customized occlusal stent was prepared. Baseline GCF samples were collected from both group sites before oral hygiene instructions and SRP. After drying and isolating the area, the end of the filter paper (Periopaper® strip) was inserted within the selected pocket sites. GCF was collected intrasulcularly until the absorbing paper strip became wet. The GCF volume collected by this method was 0-1.2 μl per strip. The strips suspected to be contaminated with blood and saliva were excluded from the study. The collected GCF samples were immediately transferred to sterilized Eppendorf tubes containing 100 µl phosphate buffered saline 10X solution and stored at -4ºC in the refrigerator, transported in a thermo-insulated flask having ice cubes to the biochemistry lab and stored at -40ºC to keep the loss of reactivity of biomarkers to a minimum. After the collection of GCF, all the clinical parameters were recorded. Full mouth SRP was performed within two to three days using ultrasonic and manual instruments, followed by subgingival placement of prepared liquorice gel on the same visit using 30-gauze side vent irrigation needles in the selected pocket sites at varying depths until the resistance was felt. Similarly, the prepared placebo gel was placed subgingivally in the selected pocket sites on the same visit. The sites were then covered by periodontal dressing (Coe-PakTM). Patients were given post-operative instructions and recalled after seven days for removal of periodontal dressing. Biochemical and clinical parameters were re-evaluated on the 15th day and 30th day after gel placement. Clinical pictures of Group A and Group B are shown in Figure 2 and Figure 3, respectively.

Biochemical analysis

Collected samples were centrifuged at 1000×g for 20 minutes at 2-8℃ per ELISA manufacturer protocol. Prepared GCF supernatant samples were evaluated for PGE2 using an ELISA kit, as shown in Figure 3.

Statistical analysis

Data were entered into a Microsoft Excel spreadsheet (Microsoft Corporation, Redmond, WA, USA) and checked for discrepancies using SPSS Statistics version 26.0 (IBM Corp. Released 2019. IBM SPSS Statistics for Windows, Version 26.0. Armonk, NY: IBM Corp). Analysis was performed using non-parametric tests, i.e., the Wilcoxon signed-rank test for intragroup comparison and the Mann-Whitney U test for intergroup comparison. Summarized data were presented using tables and graphs. Statistical significance was defined as a p-value ≤0.05.

## Results

A total of 17 patients, i.e., 60 sites, enrolled in the study. Out of 17 patients, nine were males and eight were females. Analysis was performed using non-parametric tests, i.e., Wilcoxon signed-rank test for intragroup comparison from baseline to 15th day, baseline to 30th day, and 15th day to 30th day and Mann-Whitney U test for intergroup comparison of clinical and biochemical parameters at baseline, 15th day, and 30th day.

Intragroup comparison

**Figure 4:**
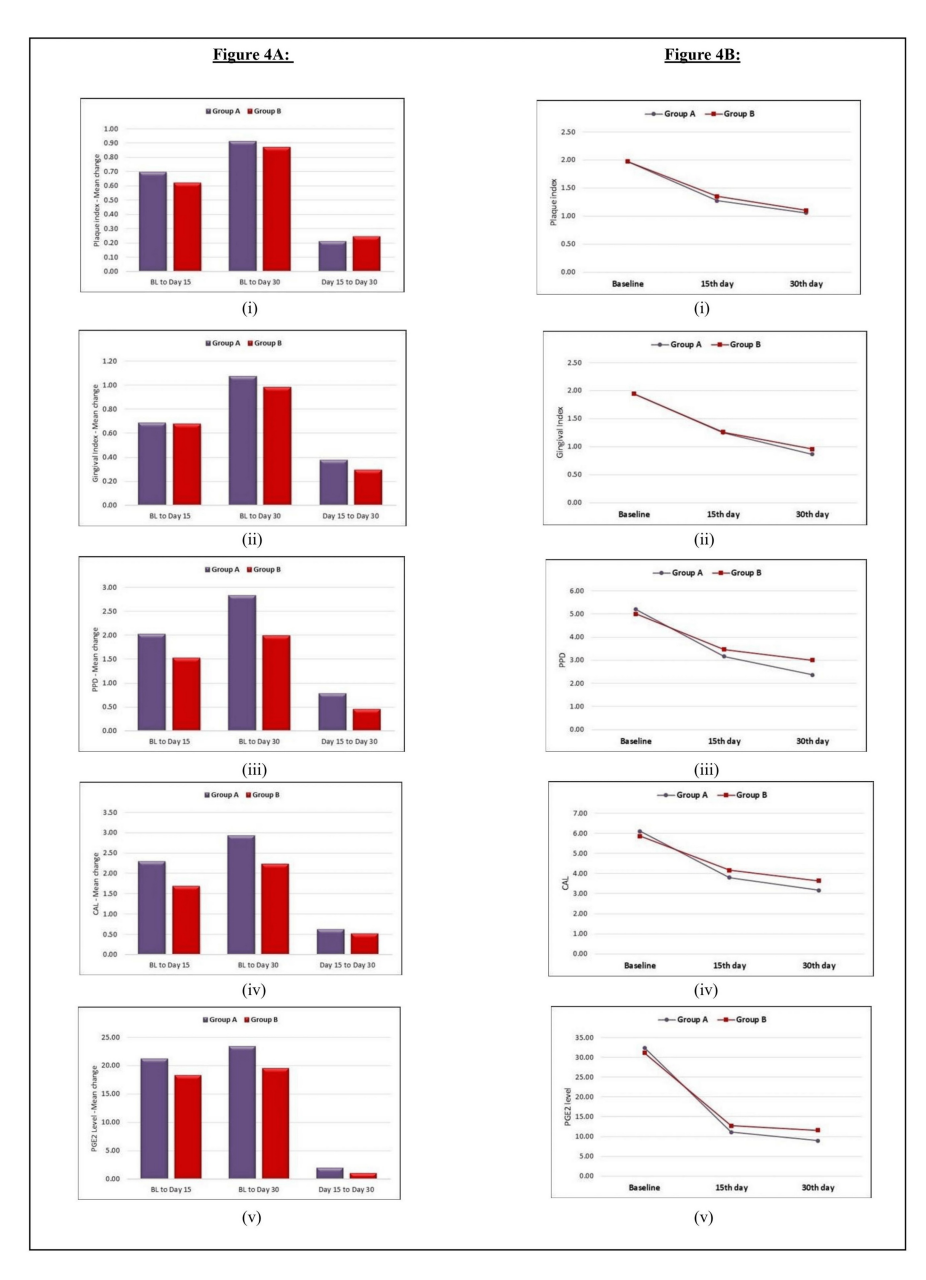
Intragroup and Intergroup comparison of clinical parameters and PGE2 levels between Group A and Group B A: (i) changes in PI, (ii) changes in GI, (iii) changes in PPD, (iv) changes in CAL, and (v) changes in PGE2 levels. B: (i) changes in PI, (ii) changes in GI, (iii) changes in PPD, (iv) changes in CAL, and (v) changes in PGE2 levels. PPD: probing pocket depth, CAL: clinical attachment level, PGE2: prostaglandin E2, PI: plaque index, GI: gingival index

The intragroup comparison of changes in clinical parameters showed a statistically highly significant difference at all time intervals (p<0.001). Similarly, on comparing PGE2 levels from baseline to the 15th day and baseline to the 30th day, a statistically highly significant reduction in both groups was seen (p<0.001). However, a non-significant reduction was noted from the 15th day to the 30th day (Table 1, Figure 4A).

**Table 1:** Intragroup comparison of changes in clinical parameters and PGE2 levels within Group A and Group B using Wilcoxon signed-rank test * Statistically highly significant (p<0.001) PPD: probing pocket depth, CAL: clinical attachment level, PGE2: prostaglandin E2, PI: plaque index, GI: gingival index

Parameters	Timeline	Group A mean ± SD	Group B mean ± SD	Group A (p-value)	Group B (p-value)
PI changes	Baseline to 15^th^ day	0.70 ± 0.25	0.63 ± 0.30	<0.001*	<0.001*
	Baseline to 30^th^ day	0.92 ± 0.22	0.88 ± 0.31	<0.001*	<0.001*
	15^th^ day to 30^th^ day	0.22 ± 0.22	0.25 ±0.23	<0.001*	<0.001*
GI changes	Baseline to 15^th^ day	0.69 ± 0.21	0.68 ± 0.25	<0.001*	<0.001*
	Baseline to 30^th^ day	1.08 ± 0.24	0.98 ± 0.24	<0.001*	<0.001*
	15^th^ day to 30^th^ day	0.38 ± 0.23	0.30 ± 0.23	<0.001*	<0.001*
PPD changes (mm)	Baseline to 15^th^ day	2.03 ± 0.49	1.53 ± 0.51	<0.001*	<0.001*
	Baseline to 30^th^ day	2.83 ± 0.46	2.00 ± 0.59	<0.001*	<0.001*
	15^th^ day to 30^th^ day	0.80 ± 0.61	0.47 ± 0.51	<0.001*	<0.001*
CAL changes (mm)	Baseline to 15^th^ day	2.30 ± 0.65	1.70 ± 0.79	<0.001*	<0.001*
	Baseline to 30^th^ day	2.93 ± 0.78	2.23 ± 0.73	<0.001*	<0.001*
	15^th^ day to 30^th^ day	0.63 ± 0.61	0.53 ± 0.57	<0.001*	<0.001*
PGE2 levels changes (ng/µl)	Baseline to 15^th^ day	21.31 ± 28.05	18.35 ± 30.29	<0.001*	<0.001*
	Baseline to 30^th^ day	23.46 ± 30.80	19.53 ± 29.88	<0.001*	<0.001*
	15^th^ day to 30^th^ day	2.15 ± 5.97	1.17 ± 6.12	0.102	0.441

Intergroup comparison

The intergroup comparison of the PI and GI between Group A and Group B was statistically non-significant at all time points as it was a split-mouth study. However, on comparing PPD, CAL, and PGE2 levels between both the groups on the 30th day, a statistically significant difference was noted (p<0.001, p=0.031, and p=0.011, respectively), with a statistically non-significant difference at baseline and 15th day, indicating that Group A exhibited a greater reduction in PPD, PGE2 levels, and gain in CAL as compared to Group B at the end of the study period (Table 2, Figure 4B).

**Table 2:** Intergroup comparison of clinical parameters and PGE2 levels between Group A and Group B using Mann-Whitney U test * statistically significant (p<0.05), * statistically highly significant (p<0.001) PPD: probing pocket depth, CAL: clinical attachment level, PGE2: prostaglandin E2, PI: plaque index, GI: gingival index

Parameters	Time points	Group A mean ± SD	Group B mean ± SD	Group A v/s Group B (p-value)
PI	Baseline	1.98 ± 0.27	1.98 ± 0.26	0.975
	15^th^ day	1.28 ± 0.21	1.35 ± 0.23	0.192
	30^th^ day	1.06 ± 0.18	1.10 ± 0.17	0.432
GI	Baseline	1.94 ± 0.16	1.94 ± 0.16	1.000
	15^th^ day	1.25 ± 0.24	1.26 ± 0.25	0.889
	30^th^ day	0.87 ± 0.25	0.96 ± 0.23	0.125
PPD (mm)	Baseline	5.20 ± 0.41	5.00 ± 0.45	0.085
	15^th^ day	3.17 ± 0.53	3.47 ± 0.63	0.053
	30^th^ day	2.37 ± 0.49	3.00 ± 0.53	<0.001*
CAL (mm)	Baseline	6.10 ± 0.99	5.87 ± 1.07	0.270
	15^th^ day	3.80 ± 1.10	4.17 ± 1.05	0.165
	30^th^ day	3.17 ± 0.83	3.63 ± 0.81	0.031*
PGE2 levels (ng/µl)	Baseline	32.43 ± 30.38	31.10 ± 30.11	0.894
	15^th^ day	11.13 ± 4.61	12.74 ± 4.64	0.156
	30^th^ day	8.98 ± 6.02	11.57 ± 4.55	0.011*

## Discussion

The initiation of periodontal disease requires a pathogen of virulent strain and a host with the necessary genetic factors to trigger the disease. The host response to pathogenic microorganisms involves a sophisticated interplay of cellular and humoral components of the immune system, which interact with each other. Host response is orchestrated by various biologically active compounds like cytokines that are pivotal in regulating the immune system, causing tissue destruction and healing [12]. Among various pro-inflammatory cytokines, PGE2 emerges as a particularly potent mediator found in abundance at sites of inflammation. PGE2 exerts multiple effects on different cell types in the periodontium, such as gingival fibroblasts, osteoblasts, osteoclasts, and immune cells. It triggers the release of inflammatory cytokines, such as IL-1, IL-6, TNF-α, and IL-8, by gingival fibroblasts and macrophages, which can further amplify the inflammatory response and cause tissue damage [13]. The levels of PGE2 in GCF exhibit a direct association with the presence of periodontal inflammation and impending tissue destruction [14]. Therefore, targeting PGE2 or its receptors may be a potential strategy for regulating inflammation and preventing tissue destruction in periodontitis.

It seems reasonable to combine mechanical periodontal therapy with the local delivery of chemotherapeutic agents to enhance the effectiveness of SRP, reduce patient discomfort, and optimize treatment outcomes. The usage of herbal agents for the treatment of periodontal disease is considered an intriguing alternative to conventional antibiotics due to their higher benefit-to-lower risk ratio. Natural products like neem, propolis, curcumin, etc. are preferred because they are safe, economical, and more effective. [15] Notably, liquorice is one such herb that is derived from dried, unpeeled stolons and roots of *Glycyrrhiza uralensis* and *Glycyrrhiza glabra*. Glycyrrhetinic acid, a constituent of liquorice extract, resembles the structure of glucocorticoids, thereby exhibiting steroid-like anti-inflammatory activity. This is due to inhibition of phospholipase A2 activity, an enzyme crucial in various inflammatory processes [16]. Hasan et al. [17] found that liquorice diminishes the synthesis of nitric oxide, PGE2, and IL-6 in lipopolysaccharide-induced macrophage cells. Bodet et al. [18] documented that liquorice extract exhibits robust anti-inflammatory properties by inhibiting the periodontopathogen LPS-induced IL-1β, IL-6, IL-8, and TNF-α responses of macrophages.

Henceforth, the present study aimed at knowing the anti-inflammatory effect of subgingivally delivered liquorice gel when used as an adjunct to SRP in inflamed periodontal tissues in chronic periodontitis patients by measuring changes in clinical parameters and PGE2 levels in GCF.

The liquorice and placebo gel used in this study were prepared as per the protocols specified by Khan et al. [19] To increase the substantivity of liquorice at the target site, Carbopol was employed as a carrier to deliver the antimicrobial agent in a controlled manner and increase its substantivity. Carbopols can expand to 1000 times their original size in water to form a large adhesive area with mucin [20]. The gel becomes thicker with the swelling of the polymer, limiting water penetration and resulting in controlled drug release.

The minimum inhibitory concentration (MIC) of glycyrrhetinic acid for periodontopathogens was estimated to be 8 mg/L to 16 mg/L. [21] Therefore, in the present study, liquorice gel was prepared with a concentration of 0.1% liquorice extract to achieve a MIC of 16 mg/L. the optimal efficacy of liquorice is observed within 15 days, with the sustained effect lasting up to 30 days. Later, the overlapping effect of oral hygiene as a confounding factor is observed. Thus, data collection intervals were set at baseline, 15th day, and 30th day after gel placement [9].

Statistical analysis has shown a significant reduction in PI and GI over time in both groups. This improvement could result from SRP aiming to reduce the microflora and their toxic products and the antimicrobial and anti-inflammatory properties of liquorice gel. These findings were similar to other studies by Ali and Mohammed [22], who also found a decrease in PI and GI over time in SRP with liquorice mouth rinse than SRP alone. Similarly, Sharma et al. [23] and Jain et al. [24] also found a decrease in GI score post-treatment with liquorice mouth rinse in both groups. Furthermore, Madan et al. [25] found a similar result regarding a decrease in gingival inflammation using *Glycyrrhiza glabra* gum paint.

The results of PPD and CAL were statistically significant in both groups at each time interval. On comparing the two groups, the results were statistically significant, with a significantly greater reduction observed in Group A than in Group B on the 30th day. Similarly, Shivprasad et al. [9] also found more PPD reduction and CAL gain in the liquorice group than in the control group.

The reduction in PGE2 levels on the 30th day could be due to the inhibitory effect of liquorice on COX-2, which results in decreased synthesis of PGE2 [4]. As discussed, liquorice is believed to show its maximum potency by 15 days, and the effect continues till 30 days. Here also, the reduction in inflammation, both clinically and biochemically, was appreciated on the 30th day.

Limitations and future prospects

The limitation of the current study is the small sample size, which can reduce its generalizability. The subgingival placement of gels may result in inappropriate retention of the drug for the desired period of time in the selected sites. Prolonged follow-up intervals are required to establish the outcome of this study conclusively.

Choi [26] indicated the enhancement of osteoblast function by glabridin (a constituent of liquorice), preventing osteoporosis and inflammatory bone diseases by reducing TNF-α-induced production of PGE2 and nitric oxide in osteoblasts. Further studies can be conducted to evaluate the role of liquorice in bone regeneration and regulation of localized bone destruction associated with inflammatory bone diseases, such as rheumatoid arthritis.

## Conclusions

The findings of the present study conclude that subgingivally delivered liquorice gel can potentially reduce inflammation in inflamed periodontal tissues. Therefore, it can be used as an anti-inflammatory agent to provide an adjunctive effect to SRP in the treatment of chronic periodontitis. Also, as an herbal agent, it is a cost-effective, easily available new treatment modality with minimum adverse effects that can be considered in daily practice. Therefore, liquorice gel can be used as an effective local drug delivery agent for the treatment of chronic periodontitis. To establish this fact, further research on a larger sample size is required to better understand its specific role in the management of periodontitis.
